# Notch Signaling in Inflammation-Induced Preterm Labor

**DOI:** 10.1038/srep15221

**Published:** 2015-10-16

**Authors:** Mukesh K. Jaiswal, Varkha Agrawal, Sahithi Pamarthy, Gajendra K. Katara, Arpita Kulshrestha, Alice Gilman-Sachs, Kenneth D. Beaman, Emmet Hirsch

**Affiliations:** 1 Department of Microbiology and Immunology, Rosalind Franklin University of Medicine and Science, North Chicago, IL, USA; 2 Department of Obstetrics and Gynecology, NorthShore University Health System, Evanston, IL; 3 Department of Obstetrics and Gynecology, Pritzker School of Medicine, University of Chicago, Chicago, IL.

## Abstract

Notch signaling plays an important role in regulation of innate immune responses and trophoblast function during pregnancy. To identify the role of Notch signaling in preterm labor, Notch receptors (Notch1-4), its ligands (DLL (Delta-like protein)-1/3/4), Jagged 1/2) and Notch-induced transcription factor Hes1 were assessed during preterm labor. Preterm labor was initiated on gestation day 14.5 by intrauterine (IU) injection of peptidoglycan (PGN) and polyinosinic:cytidylic acid (poly(I:C). Notch1, Notch2, Notch4, DLL-1 and nuclear localization of Hes1 were significantly elevated in uterus and placenta during PGN+poly(I:C)-induced preterm labor. *Ex vivo*, Gamma secretase inhibitor (GSI) (inhibitor of Notch receptor processing) significantly diminished the PGN+poly(I:C)-induced secretion of M1- and M2-associated cytokines in decidual macrophages, and of proinflammatory cytokines (IFN-γ, TNF-α and IL-6) and chemokines (MIP-1β) in decidual and placental cells. Conversely, angiogenesis factors including Notch ligands Jagged 1/2 and DLL-4 and VEGF were significantly reduced in uterus and placenta during PGN+poly(I:C)-induced preterm labor. *In vivo* GSI treatment prevents PGN+poly(I:C)-induced preterm delivery by 55.5% and increased the number of live fetuses *in-utero* significantly compared to respective controls 48 hrs after injections. In summary, Notch signaling is activated during PGN+poly(I:C)-induced preterm labor, resulting in upregulation of pro-inflammatory responses, and its inhibition improves *in-utero* survival of live fetuses.

Notch signaling is evolutionarily conserved and critical for development and homeostasis in various tissues^1^,^2^. Notch signaling pathways exert effects throughout the pregnancy, playing an important role in placental angiogenesis and trophoblast function^3^. Notch receptors operate both on the cell surface to receive activating signals and within the nucleus as transcriptional modulators. The core mammalian Notch signaling pathway consists of a conserved family of four transmembrane receptors (Notch1-4) and five ligands (DLL (Delta-like protein)-1/3/4 and Jagged 1/2). Binding of receptors and ligands on adjacent cells triggers serial proteolytic cleavage of the receptor, releasing the Notch intracellular domain (NICD) via γ-secretase mediated processing. Subsequently, cleaved NICD translocates to the nucleus, binds to transcription factors, and induces downstream targets^4^.

Evidence suggests that there is cross-talk between Notch and toll-like receptor (TLR) signaling pathways^5^,^6^. Notch signaling plays a crucial role in macrophage polarization, promoting the M1 (inflammatory) subtype over the M2 (anti-inflammatory) subtype^7^. TLR activation up-regulates the expression of Notch ligands and receptors, favoring the activation of Notch signaling, and amplifies the inflammatory response by enhancing NF-κB signaling^8^. For example, lipopolysaccharide (LPS, a TLR4 ligand) activates Notch signaling through a JNK-dependent pathway that subsequently regulates the inflammatory response^9^. Notch and TLR signaling pathways cooperate to activate the transcription of Notch target genes, including transcription factors Hes1 (hairy and enhancer of split-1, a canonical Notch target and transcriptional factor responsible for sustaining NF-κB activation^8^) and Hey1 (hairy/enhancer-of-split related with YRPW motif protein 1). This leads to increased production of TLR-triggered cytokines such as TNF-α, IL-6, and IL-12^10^. Several studies also indicate that Notch signaling plays an important role in inflammatory disorders^11^,^12^.

Notch1 signaling is reported to modulate multiple signaling mechanisms crucial for decidualization in the artificial decidualization model in mice^13^ and in primates^14^, which is essential for the establishment of a successful pregnancy. Decreased Notch signaling is also reported to be associated with endometriosis and impaired decidualization in human^15^. Defects of Jagged 1 and DLL-4 in placental trophoblast causes abnormal placental angiogenesis^3^, which contributes to pregnancy complications, such as pre-eclampsia^4^,^16^.

Preterm birth is one of the most significant causes of neonatal mortality and morbidity. About 40% of cases of preterm labor are associated with infection within the gestational compartment^17^,^18^. We and others have shown that preterm labor can be induced in animal models by pathogen-derived TLR ligands for TLR4 (LPS^19^), TLR2 (peptidoglycan, PGN), TLR3 (polyinosinic:cytidylic acid, poly(I:C))^20^, and in a synergistic manner, TLR2+TLR3^19^,^21^,^22^,^23^. The combination of PGN+poly(I:C) (used in the present study) has a synergistic effect on preterm labor and leads to 100% preterm delivery when compared to the same doses of PGN (22% preterm delivery) or poly(I:C) (14% preterm delivery) alone^23^. This combination of PGN+poly(I:C) induces the preterm labor via simultaneous activation of apoptosis and inflammatory processes^24^. Such combined stimulation of TLR2 and TLR3 receptors results in simultaneous activation of both known TLR downstream signaling pathways, known as the MyD88 (myeloid differentiation primary response gene 88)-dependent and the MyD88-independent pathways. Activation of these pathways mimics clinical infection in certain scenarios, such as 1) engagement of TLR4 by Gram negative bacteria or viral/bacterial super-infection^25^; 2) activation of both TLR3 and another TLR simultaneously by a single organism (e.g., murine cytomegalovirus, herpes simplex virus, and Schistosoma mansoni^26^,^27^); 3) superinfection, in which a host is infected simultaneously by more than one microorganism, such as a virus and a bacterium^25^; and 4) activation of TLRs by one of several known, endogenously produced TLR ligands together with an exogenous pathogen^28^,^29^.

We hypothesized that Notch signaling is an important factor in the regulation of pregnancy and may be involved, in part, in inflammation-induced preterm labor. In the current study, we determined the role of Notch signaling in PGN+poly(I:C)-induced preterm labor in the mouse and characterized its association with inflammation. We found that Notch ligand (DLL-1), its receptors (Notch1, 2 and 4), and the transcription factor Hes1 were significantly elevated during PGN+poly(I:C)-induced preterm labor. Conversely, Notch ligands DLL-4, Jagged 1 and Jagged 2, which are involved in angiogenesis, were significantly suppressed during PGN+poly(I:C)-induced preterm labor. Suppression of Notch signaling *ex vivo* using gamma secretase inhibitor (GSI) significantly diminished PGN+poly(I:C)-induced inflammation and also reduced the secretion of VEGF. These distinct opposing effects of PGN+poly(I:C) on inflammation-associated Notch ligand (DLL-1) and angiogenesis-associated Notch ligands (DLL-4, Jagged 1 and 2) signify that Notch signaling pathways are modulated bidirectionally during PGN+poly(I:C)-induced preterm labor. Instead of its bidirectional effect, GSI treatment was able to increase *in-utero* survival of the fetuses and prevents PGN+poly(I:C)-induced preterm delivery by 55.5%.

## Results

### DLL-1 ligand increases in decidual macrophages during PGN+poly(I:C)-induced preterm labor

Notch signaling is activated in response to toll-like receptor (TLR) ligands, thus amplifying the inflammatory response by enhancing NF-κB signaling^8^. Therefore, to identify the role of Notch signaling during preterm labor induced by TLR ligands, the expression of Notch ligand (DLL-1), its receptors (Notch1, 2, 3 and 4) and the transcription factor Hes1 were assessed at the feto-maternal interface during preterm labor after intrauterine administration of PGN+poly(I:C) in mice^19^,^23^. Uteri and placentas (from regions inclusive of the decidual caps underlying placental attachment sites) were harvested 8 h after surgery.

**Figure 1:**
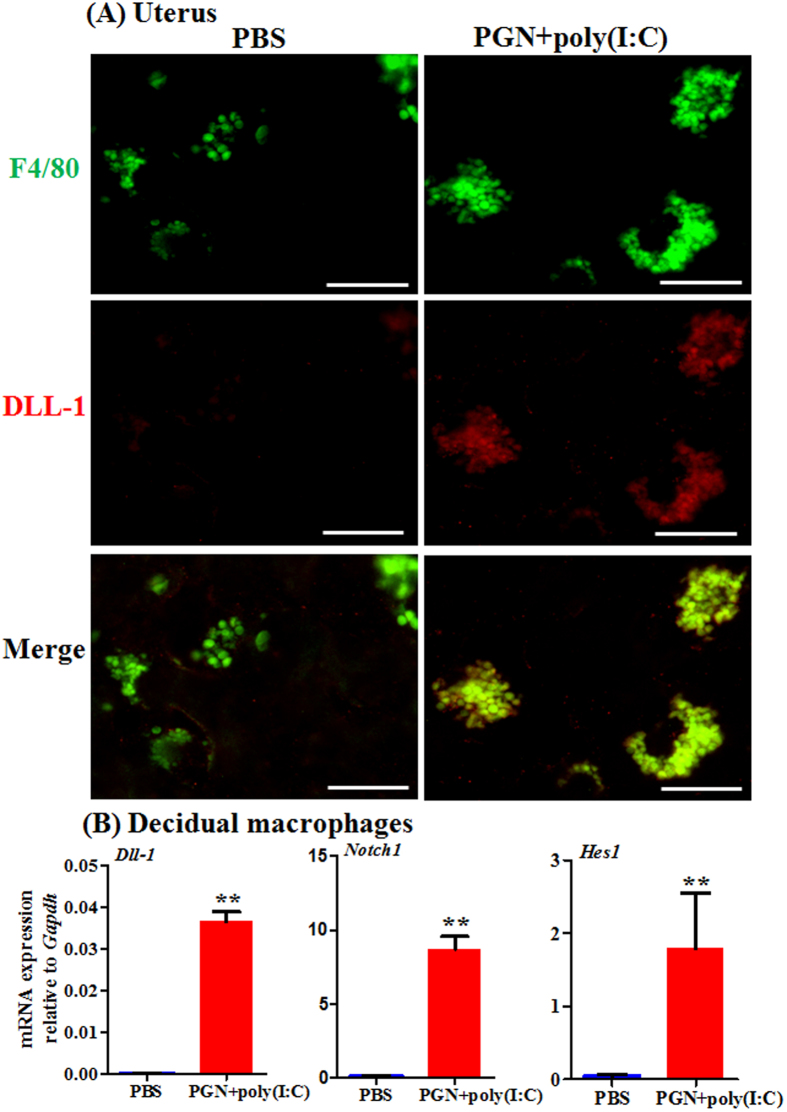
DLL-1 ligand, Notch1 and Hes1 increase in decidual macrophages during PGN+poly(I:C)-induced preterm labor. (**A**) Co-localization of macrophages stained with F4/80 (green) and DLL-1 (red), and merged images of F4/80 and DLL-1 in uterine decidua from mice treated with either PBS or PGN+poly(I:C). N = 4-5 animals per group. Six sections per animal were analyzed. Original magnification: 400 X. Bars: 10 μm (**B**) The mRNA expression of DLL-1, Notch1 and Hes1 in decidual macrophages recovered from mice on day 14.5 of normal pregnancy, cultured *ex vivo* and treated with PBS or PGN+poly(I:C) for 2 h. N = 3 each group. Error bars = ±SEM. **P ≤ 0.01 Significant difference vs. PBS.

Macrophages are considered a critical cell type responsible for labor. They infiltrate gestational tissues during preterm labor induced by inflammation^24^,^30^. Therefore we studied the role of Notch signaling in decidual macrophages during PGN+poly(I:C)-induced preterm labor. Double immunofluorescence staining of F4/80 (a macrophage marker) and DLL-1 ligand shows that PGN+poly(I:C) induces DLL-1 ligand in decidual macrophages (Fig. 1A). The uteroplacental unit is shown with the demarcation of different zones (Supplemental Fig. 1A). Isotype controls are also shown (Supplemental Fig. 2). We further explored Notch signaling in decidual macrophages obtained from mice on day 14.5 of normal pregnancy and cultured *ex vivo*. The mRNA expression of DLL-1 ligand, Notch1 and transcription factor Hes1 was significantly higher in decidual macrophages treated with PGN+poly(I:C) as compared to control (Fig. 1B).

### Inhibition of Notch signaling suppresses inflammatory responses in decidual macrophages

**Figure 2:**
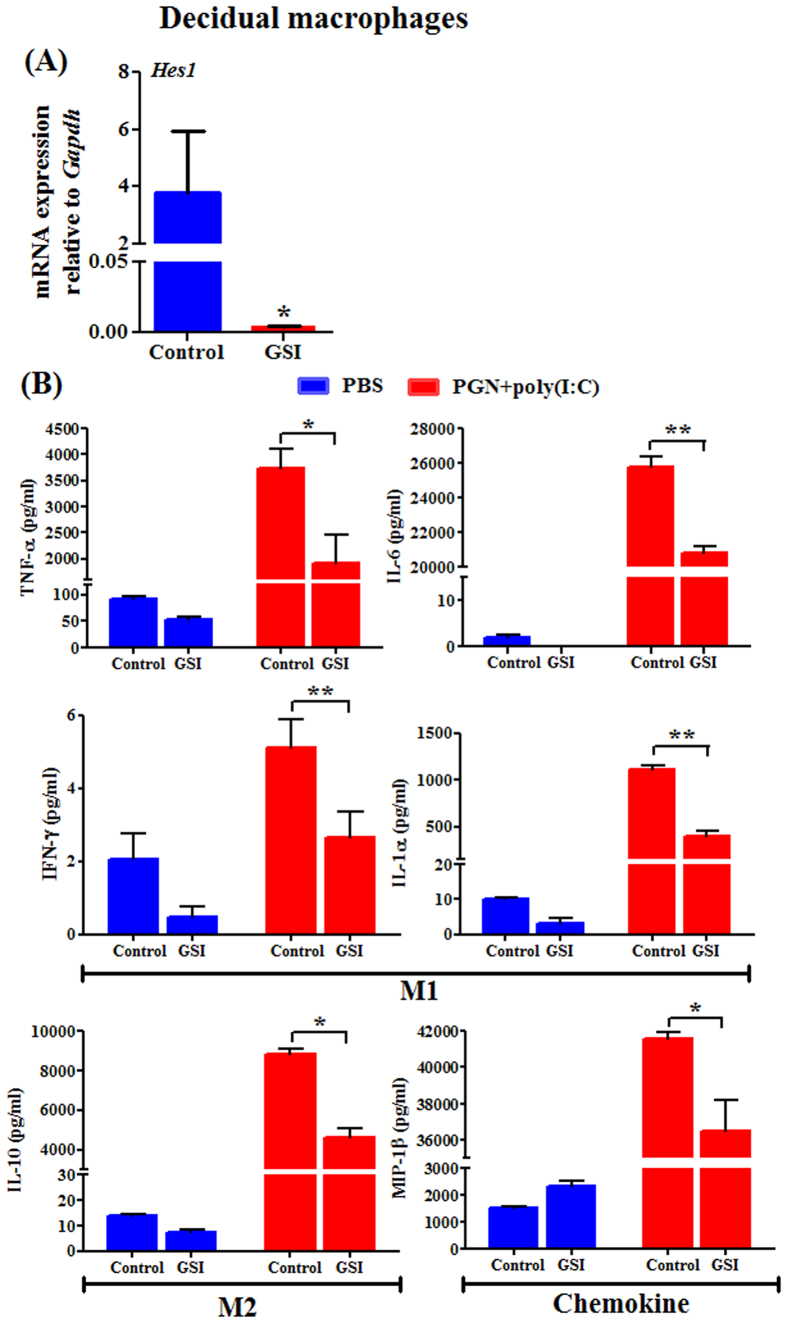
Inhibition of Notch signaling suppresses inflammatory responses in decidual macrophages. (**A**) GSI (gamma secretase inhibitor, an inhibitor of Notch receptor processing) treatment suppresses Hes1 mRNA expression which shows the effect of GSI on Notch signaling in decidual macrophages. *P ≤ 0.05 Significant difference vs. control. (**B**) M1 and M2 macrophage profile associated cytokines and chemokine were measured by Luminex assay in medium from decidual macrophages recovered from mouse on day 14.5 of normal pregnancy, cultured *ex vivo* and treated with PBS and PGN+poly(I:C) for 2 h, followed by treatment with either control or GSI for 10 h. N = 3 each group. Error bars = ± SEM. *P ≤ 0.05, **P ≤ 0.01 Significant difference between PGN+poly(I:C) treated with control/GSI.

Previously we have shown that PGN+poly(I:C)-induced preterm labor directs decidual macrophages toward a double-positive phenotype consisting of both M1 markers (CD11c+) and M2 markers (CD206+)^24^. Activation of Notch signaling promotes the polarization of macrophages to the IFN-γ producing M1 macrophage subtype^7^. Chemical inhibitors of Notch signaling like gamma secretase inhibitor (GSI) (an inhibitor of Notch receptor processing) are currently in clinical trials for inflammatory diseases like Alzheimer’s disease and cancer^12^,^31^. Inhibition of Notch signaling by GSI reduces NF-κB activity and suppresses inflammatory responses^8^,^9^,^32^. Given the importance of Notch signaling in macrophage polarization, we used GSI to study its effect on PGN+poly(I:C)-induced cytokines profile in decidual macrophages. We confirmed that GSI treatment in decidual macrophages suppress the Notch signaling by the decreased mRNA expression of Hes1 (Fig. 2A). The Notch signaling inhibitor GSI significantly decreases PGN+poly(I:C)-induced secretion of M1- (TNF, IL-6, IFN-γ and IL-1α) and M2- (IL-10) associated cytokines as well as the chemokine MIP-1β from decidual macrophages (Fig. 2B). These data provide evidence that Notch signaling is important for generating the double-positive M1^+^M2^+^ decidual macrophage phenotype which is critical during PGN+poly(I:C)-induced preterm labor.

### DLL-1 ligand and Notch receptor increases in the uterus and placenta during PGN+poly(I:C)-induced preterm labor

**Figure 3:**
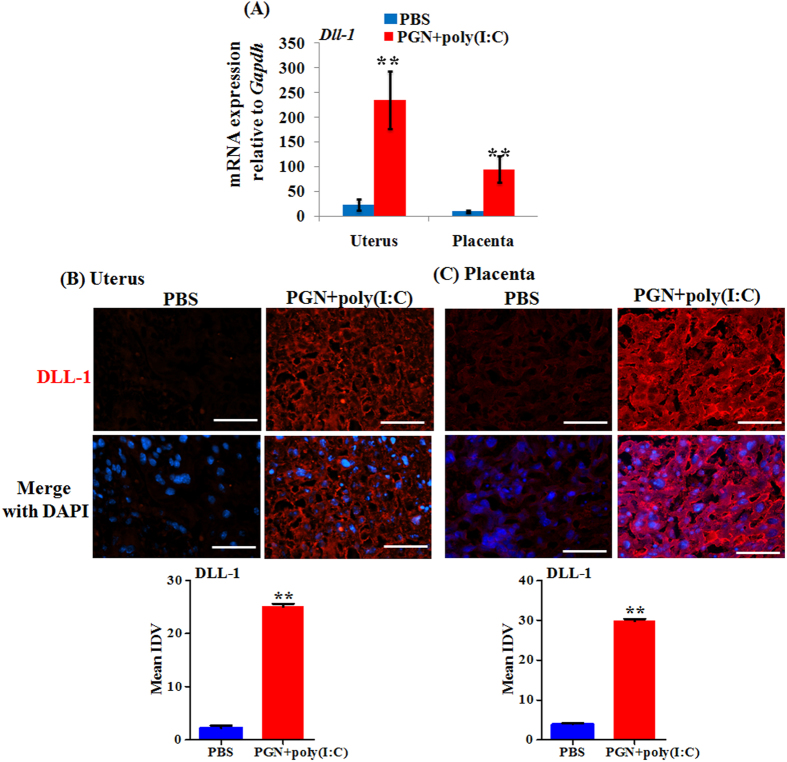
DLL-1 ligand is increased during PGN+poly(I:C)-induced preterm labor. (**A**) The mRNA expression of DLL-1 in uterus and placenta recovered from PBS and PGN+poly(I:C) treated groups. N = 6–11 each group. Immunofluorescence staining (Upper panel) and mean integrated density value (IDV) (lower panel) of DLL-1 (red) in uterus (**B**) and placenta (**C**) from PBS and PGN+poly(I:C) treated groups. Nuclei stained with DAPI (blue) in merged images. N = 4–5 each group. Six sections per animal were analyzed. Original magnification: 200 X. Bars: 10 μm. PBS and PGN+poly(I:C): intrauterine injections on day 14.5. Error bars = ±SEM. **P ≤ 0.01 Significant difference vs. PBS.

The mRNA expression of DLL-1 ligand was significantly increased during PGN+poly(I:C)-induced preterm labor in uterus and placenta (Fig. 3A). Immunofluorescence staining correlated with mRNA levels and showed that expression of DLL-1 was increased in both uterine decidua and placenta (Fig. 3B,C).

**Figure 4:**
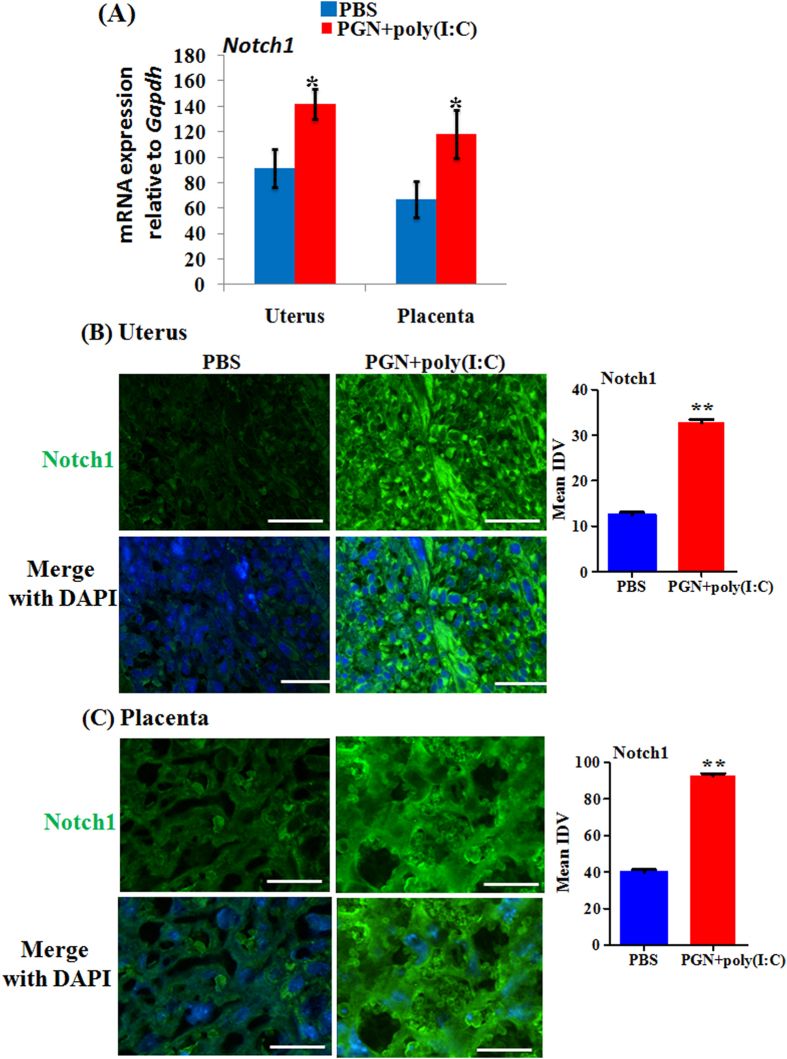
Notch receptor increases during PGN+poly(I:C)-induced preterm labor. (**A**) The mRNA expression of Notch1 in uterus and placenta recovered from PBS and PGN+poly(I:C) treated groups. N = 6–11 each group. Immunofluorescence staining and mean integrated density value (IDV) of Notch1 (green) in uterus (**B**) and placenta (**C**), nuclei stained with DAPI (blue) in merged images. N = 4–5 each group. Six sections per animal were analyzed. Original magnification: 200 X. Bars: 10 μm. PBS and PGN+poly(I:C): intrauterine injections on day 14.5. Error bars = ±SEM. *P ≤ 0.05 **P ≤ 0.01 Significant difference vs. PBS.

**Figure 5:**
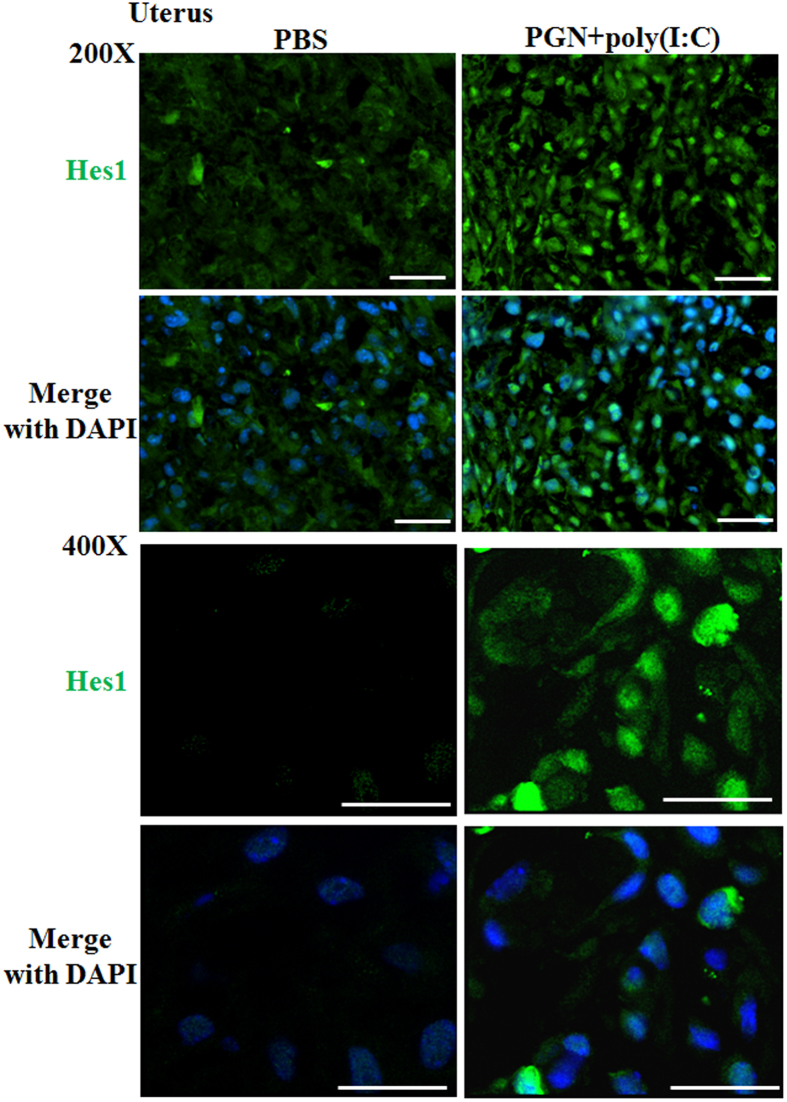
Hes1 expression during PGN+poly(I:C)-induced preterm labor. Immunofluorescence staining of Hes1 (green) in uterus from PBS and PGN+poly(I:C) treated groups. Nuclei stained with DAPI (blue) in merged images. N = 4–5 each group. Six sections per animal were analyzed. Original magnification: 200 X and 400X. Bars: 10 μm. PBS and PGN+poly(I:C): intrauterine injections on day 14.5.

The mRNA expression of Notch1 was significantly increased during PGN+poly(I:C)-induced preterm labor in uterus and placenta (Fig. 4A). Immunofluorescence staining confirms this expression pattern on the protein level (Fig. 4B,C). Notch2 and 4 were increased in placenta (Supplemental Fig. 3). Notch3 mRNA expression was undetectable in both the uterus and placenta. The protein levels of Hes1 were higher with more nuclear localization in uterine decidua (Fig. 5) and placenta (data not shown) with PGN+poly(I:C) treatment. Isotype controls are also shown (Supplemental Fig. 2).

Taken together, these results suggest that Notch signaling is activated during PGN+poly(I:C)-induced preterm labor. Activation of Notch signaling has been shown to be involved in the sustained activation of NF-κB and enhancement of inflammatory responses^8^,^9^,^32^. Therefore, we further examined the involvement of Notch signaling in inflammation induced by PGN+poly(I:C) treatment at the feto-maternal interface.

### Inhibition of Notch signaling suppresses the inflammatory response in decidual and placental cells

**Figure 6:**
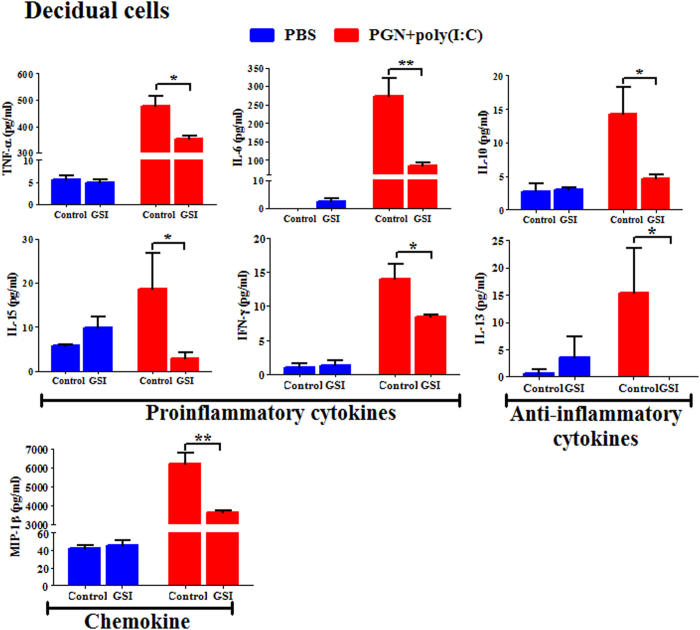
Inhibition of Notch signaling suppresses inflammatory responses in decidual cells. Pro-inflammatory and anti-inflammatory cytokines and chemokine were measured by Luminex assay in protein extracted from decidual cells recovered from mouse on day 14.5 of pregnancy, cultured *ex vivo* and treated with PBS and PGN+poly(I:C) for 2 h, followed by treatment with either control or GSI for 10 h. N = 3 each group. Error bars =±SEM. *P ≤ 0.05, **P ≤ 0.01 Significant difference between PGN+poly(I:C) treated with control/GSI.

To study the role of Notch signaling in preterm labor in the context of inflammation, decidual single cell suspensions were prepared from mouse tissues obtained on day 14.5 of pregnancy. Cells were cultured *ex vivo* and incubated with PGN+poly(I:C) or PBS for 2 h, followed by treatment with GSI or control for 12 h. GSI significantly decreased PGN+poly(I:C)-induced secretion of proinflammatory cytokines (TNF, IL-6, IL-15 and IFN-γ), anti-inflammatory cytokines (IL-10 and IL-13) and also chemokine (MIP-1β) (Fig. 6). Overall, similar findings were obtained in placental cells (Supplemental Fig. 4).

### Expression of angiogenesis-associated Notch ligands decreases during PGN+poly(I:C)-induced preterm labor

**Figure 7:**
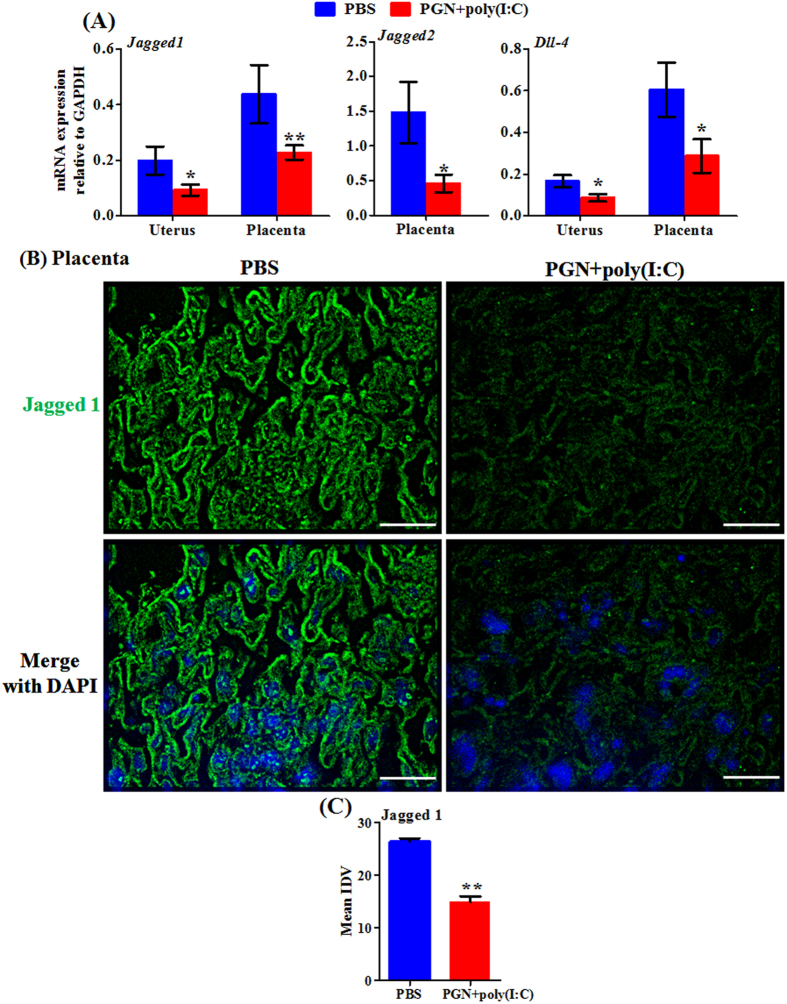
Angiogenesis-associated Notch ligands decrease during PGN+poly(I:C)-induced preterm labor. (**A**) The mRNA expression of Jagged 1, Jagged 2 and DLL-4 in uterus and placenta recovered from PBS and PGN+poly(I:C) treated groups. N = 6–11 each group. (**B**) Immunofluorescence staining and (**C**) mean integrated density value (IDV) of Jagged 1 (green) in placenta. Nuclei stained with DAPI (blue) in merged images. N = 4–5 each group. Six sections per animal were analyzed. Original magnification: 200 X. Bars: 10 μm. PBS and PGN+poly(I:C): intrauterine injections on day 14.5. Error bars = ±SEM. *P ≤ 0.05, **P ≤ 0.01 Significant difference vs. PBS.

Apart from the regulation of inflammation, another important function of Notch signaling is angiogenesis^33^. Placental vascularization and angiogenesis are critical for the development of viable healthy offspring^34^. A decreased level of angiogenic factor VEGF in placenta causes reduction in placental vascularization during late pregnancy and leads to restricted fetal growth and poor pregnancy outcomes^35^. Notch signaling mediated by DLL-4, Jagged 1 and 2 is indispensable for vascular development during fetal development^33^,^36^,^37^. Therefore, we measured the expression of the above angiogenesis-associated Notch ligands during PGN+poly(I:C)-induced preterm labor. The mRNA expression of Jagged 1, Jagged 2 and DLL-4 was significantly decreased in uterus and placenta during PGN+poly(I:C)-induced preterm labor, except for Jagged 2, which was undetectable in uterus (Fig. 7A). Immunofluorescence staining confirms decreased protein expression of Jagged 1 in placenta during PGN+poly(I:C)-induced preterm labor (Fig. 7B).

**Figure 8:**
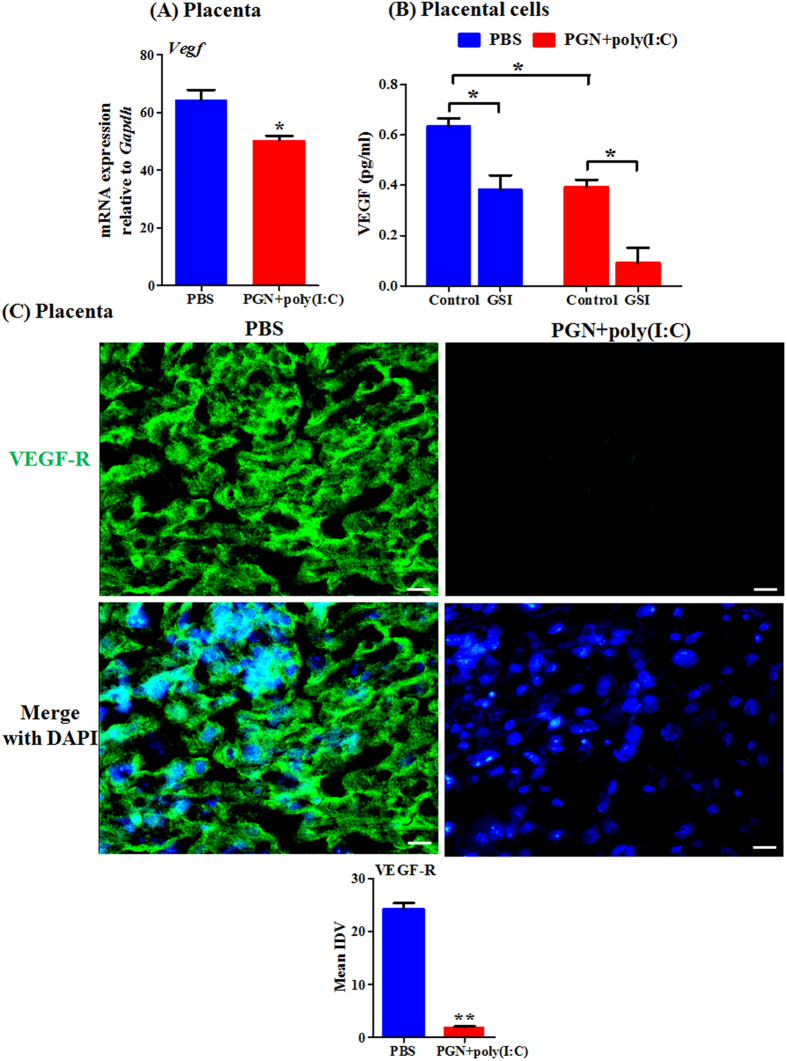
VEGF and its receptor decrease during PGN+poly(I:C)-induced preterm labor and inhibition of Notch signaling suppresses VEGF secretion. (**A**) The mRNA expression of VEGF in placenta recovered from PBS and PGN+poly(I:C) treated groups. N = 6–11 each group. *P ≤ 0.05 Significant difference vs. PBS (**B**) Protein concentration of VEGF measured by Luminex assay in protein extracted from placental cells recovered from mouse on day 14.5 of pregnancy, cultured *ex vivo* and treated with PBS and PGN+poly(I:C) for 2 h, followed by treatment with either control or GSI for 10 h. N = 3 each group. *P ≤ 0.05 Significant difference between PGN+poly(I:C) treated with control/GSI. (**C**) Immunofluorescence staining (Upper panel) and mean integrated density value (IDV) (lower panel) of VEGF-R (green) in placenta. Nuclei stained with DAPI (blue) in merged images. N = 4-5 each group. Six sections per animal were analyzed. Original magnification: 200 X. Bars: 10 μm. PBS and PGN+poly(I:C): intrauterine injections on day 14.5. Error bars = ±SEM. **P ≤ 0.01 Significant difference vs. PBS.

The vascular endothelial growth factors (VEGFs) are other critical angiogenic factors regulated by Notch signaling mediators^34^,^38^. Therefore, we checked the expression of VEGF during PGN+poly(I:C)-induced preterm labor. The mRNA expression of VEGF was decreased in placenta during PGN+poly(I:C)-induced preterm labor (Fig. 8A). PGN+poly(I:C) treatment in *ex vivo* cultured placental cells significantly decreases VEGF secretion in comparison to PBS. Furthermore, GSI treatment in *ex vivo* cultured placental cells also significantly decreases VEGF secretion with further decrease in the presence of PGN+poly(I:C) (Fig. 8B).

The protein expression of VEGF-receptor (VEGF-R) was also checked during PGN+poly(I:C)-induced preterm labor. Immunofluorescence staining shows that protein expression of VEGF-R was decreased in placenta during PGN+poly(I:C)-induced preterm labor (Fig. 8C).

### Effect of GSI on PGN+poly(I:C)-induced preterm delivery

Based on the observed findings above, we explored the use of GSI for the treatment of PGN+poly(I:C)-induced preterm delivery. A single dose of GSI (300 μg) was injected IU immediately after PGN+poly(I:C) IU injection on day 14.5 of pregnancy in mice. As shown in supplemental Table 1, GSI treatment was able to prevent PGN+poly(I:C)-induced preterm delivery by 55.5%. Moreover, the number live fetuses *in-utero* was also increased (7.9 ± 1.23) significantly compared to respective control (0.9 ± 0.62) 48 hrs after injections.

## Discussion

Notch signaling plays a critical role in decidualization^14^,^39^, spiral artery remodeling^40^ and placental development during pregnancy^3^. Specifically, DLL-1 is expressed on leukocytes and interacts with Notch receptors to induce IFN-γ, which leads to vascular smooth muscle disruption, a process required for proper trophoblast invasion^40^,^41^. Notch ligands DLL-4, Jagged 1 and 2 are expressed in decidual and trophoblast cells and are involved in angiogenesis during placental vascularization via the secretion of growth factors such as VEGF^41^,^42^. Our study focuses on the role the Notch signaling during inflammation-induced parturition.

Various reports have shown that proinflammatory factors not only polarize macrophages to the M1 type but are also associated with the upregulation of Notch pathway molecules, which leads to canonical Notch signaling activation^7^,^43^. Previously we have shown that upon PGN+poly(I:C) treatment decidual macrophages became double-positive for CD11c (M1 marker) and CD206 (M2 marker), which leads to the secretion of both M1-associated cytokines (INF-γ, IL-6, TNF) and the M2-associated cytokine (IL-10). We now add the new findings of PGN+poly(I:C)-induced expression of DLL-1 and Notch1 in decidual macrophages and of suppression of the secretion of both M1- and M2-assocatied cytokines by the Notch inhibitor gamma-secretase inhibitor (GSI). These findings suggest that Notch signaling mediates PGN+poly(I:C)-induced decidual macrophages polarization.

Activation of Notch signaling enhances the inflammatory response by increasing the NF-κB activity^32^. Notch ligand DLL-1 is associated with secretion of IFN-γ from the macrophages and blocking of Notch signaling by GSI decreased the levels of proinflammatory cytokines like IFN-γ^10^,^44^. We found that Notch signaling is activated during PGN+poly(I:C)-induced preterm labor as shown by increased expression of DLL-1, Notch1 and higher nuclear translocation of Hes1 in the decidual and placental cells. On the one hand, there is induction of a robust pro-inflammatory cytokine profile in decidual and placental cells, an event suppressed by GSI, a Notch inhibitor.

This study also showed that, the angiogenesis specific Notch ligands like Jagged 1, Jagged 2 and DLL-4 were reduced in uterus and placenta during PGN+poly(I:C)-induced preterm labor. These ligands play a crucial role in the angiogenesis by regulating angiogenic factor VEGF^45^,^46^ and the level of VEGF is reduced in placenta during gestational hypertensive disorders and preterm birth^47^. The observed reduced level of VEGF in placenta during PGN+poly(I:C)-induced preterm labor is further reduced by GSI treatment and suggests that the Notch signaling is also critical for the regulation of angiogenesis in the placenta. Other reports also suggest that over expression of DLL-4 and Jagged 1 enhances the angiogenesis^46^,^48^ and inhibition or mutation of these genes cause abnormal angiogenesis in the placenta which leads to pre-eclampsia^4^,^16^.

Considering to target Notch signaling as a therapeutic opportunity for the treatment of preterm labor is enormously critical because of its bidirectional modulation: 1) suppression of Notch signaling by using GSI significantly diminished the PGN+poly(I:C)-induced inflammation; 2) the distinct opposing functional effects of inflammation-associated Notch ligand (DLL-1) and angiogenesis-associated Notch ligands (Jagged 1, 2 and DLL-4) need careful monitoring for the treatment of inflammation-induced preterm labor. Despite, this bidirectional effect of PGN+poly(I:C) on Notch signaling, GSI treatment was able to prevent preterm delivery by 55.5% and significantly improves *in-utero* survival of the fetuses. Thus, inhibition of Notch signaling during inflammation-induced preterm labor might be predicted to have a beneficial anti-inflammatory effect over the harmful effect on placental angiogenesis.

In summary, our data have identified novel roles for Notch signaling in PGN+poly(I:C)-induced preterm labor: 1) enhancing inflammation; 2) promoting decidual macrophage polarization; 3) diminishing angiogenic factors; 4) GSI treatment with PGN+poly(I:C) improves the number of live fetuses *in-utero*. Future challenges are to better understand the breadth of action of Notch signaling and to optimize the potential beneficial effects of Notch signaling in the prevention of preterm labor.

## Methods

### Mice

All procedures involving animals were approved by the Institutional Animal Care and Use Committee of Rosalind Franklin University of Medicine and Science, North Chicago, IL, USA and NorthShore University HealthSystem Animal Care, Evanston, IL, USA and Use Committee and conform to the Guide for Care and Use of Laboratory Animals (1996, National Academy of Sciences). Mice used in the present studies were CD-1 strain (Harlan laboratories, Madison, WI). Female mice in estrus were selected by the gross appearance of the vaginal epithelium^49^ and were impregnated naturally. Mating was confirmed by the presence of a vaginal plug, and the day of plug formation was counted as day 0.5 of pregnancy.

### Animal treatment and tissue harvest

Intrauterine (IU) injection of the combination of PGN (TLR2 agonist, extracted from *Staphylococcus aureus,* 77140, Sigma, 0.3 mg/mouse) plus poly(I:C) (a synthetic analog of double-stranded RNA and a TLR3 agonist, 27-4729-01, Amersham Biosciences, 1.0 mg/mouse) or PBS control was performed with general anesthesia and laparotomy into the right uterine horn on day 14.5 of a 19-20 day gestation period, as previously described^19^,^23^,^50^. PGN and poly(I:C) were combined because we showed previously that this combination produces dramatic synergy in both, preterm delivery (leading to delivery within 18-24 hours of treatment) and inflammatory responses^19^,^23^,^24^. The abdomen was closed in two layers, with 4-0 polyglactin sutures at the peritoneum and wound clips at the skin. Surgical procedures lasted approximately 10 minutes. Animals recovered in individual, clean cages in the animal facility.

To study effects of these treatments *in vivo*, animals were euthanized 8 h after surgery. The inoculated/right horn was incised longitudinally along the anti-mesenteric border. Uteri (from regions inclusive of the decidual caps underlying placental attachment sites) and placentas were harvested, washed in ice-cold PBS, flash-frozen in liquid nitrogen and stored at −85 °C for mRNA extraction or fixed in 10% neutral buffered formalin for immunohistochemistry.

### Extraction of decidual macrophages

Decidual macrophages were isolated as previous described by Co *et al.*^51^, with slight modifications. Decidual caps were collected on day 14.5 of pregnancy, minced gently and incubated in 50 ml of PBS containing 30U collagenase type II (Gibco, Grand Island, NY) in a shaking water bath at 37 °C for 20 min. The collagenase reaction was stopped by washing with PBS supplemented with 10% fetal calf serum. Cells were strained through a 70-μm nylon strainer to remove debris, washed with PBS and layered over 15 ml Ficoll-Paque (GE Healthcare Life Sciences, Pittsburgh, PA) and centrifuged at 1200 rpm for 20 min at 4 °C. A crude decidual leukocyte fraction was collected from the supernatant-Ficoll interface and washed twice by centrifugation in HBSS at 300 × g for 5 min. For purification of decidual macrophages F4/80^+^ macrophages were flow-sorted from the decidual leukocyte fraction using anti-F4/80-APC antibody (Biolegend, San Diego, CA) on FACS aria with FACSDiva software (BD Biosciences, San Jose, CA). Isolated decidual macrophages (4x10^5^ cells/well) were cultured in DMEM High Glucose (Gibco) supplemented with 10% fetal bovine serum, 1% streptomycin and 1% penicillin in 48-well plates at 37 °C in 5% CO_2_/95% air for 1 h prior to further treatment (see below). RAW 264.7 macrophage cells were used as a positive control for F4/80 staining (Supplemental Fig. 1B).

### Decidual and placental cell preparation

Uteri were dissected on day 14.5 of pregnancy and decidual caps and placentas were collected. Decidua and placenta were prepared as single-cell suspensions as described previously^52^. Briefly, tissues were minced in Hank’s balanced salt solution (HBSS, Life technologies, Grand Island, NY), mechanically dispersed through a 100-μm nylon filter, and centrifuged at 1500 rpm. The remaining pellet was dispersed in RPMI medium at 10^7^cells/ml in 48-well plates. Prior to plating, placental suspensions underwent red cell lysis by incubation with red blood cell lysis buffer (BioLegend) according to the manufacturer’s instructions. The above specimens were incubated at 37 °C in 5% CO_2_/95% air for 1 h prior to treatment (see below). Viability of *ex vivo* cultured cells was >95% as assessed using the trypan blue dye exclusion test.

### *Ex vivo* treatment

Decidual macrophages or decidual and placental cells were incubated for 2 h in the presence of PBS or PGN (1 μg/ml) plus poly(I:C) (10 μg/ml) followed by treatment for 10 h with either gamma secretase inhibitor (GSI, an inhibitor of Notch receptor processing, 20 μM, Millipore, Billerica, MA) or control (solvent for GSI (DMSO diluted in PBS at ~1:1300)). All experiments were conducted in triplicate and repeated twice (i.e. three triplicate experiments).

### GSI treatment *in vivo*

A 60 μl solution of GSI (300 μg/animal) or vehicle control (solvent for GSI (DMSO same volume as GSI)) was injected intrauterine (IU) simultaneously after PGN+poly(I:C) IU injection (as described above). The timing of preterm delivery and number of live and dead fetuses were observed. At necropsy the number of fetuses delivered or remaining *in utero* and the survival status of these retained fetuses (as determined by cardiac or vascular pulsations in the fetal bodies and membranes) were recorded.

### Real-time PCR

Total RNA from uteri (from regions inclusive of the decidual caps underlying placental attachment sites) and placentas was extracted after homogenization in Trizol reagent (Life technologies) according to the manufacturer’s protocol. For *ex vivo* experiments, cells were either lysed in culture dishes or cell pellets were homogenized in Trizol. cDNA was prepared using qScript cDNA super mix (Quanta Biosciences, Gaithersburg, MD). Duplex RT-PCR was performed with one primer pair amplifying the gene of interest and the other an internal reference (GAPDH) in the same tube using the Applied Biosystems Step One Real-time PCR system. Semiquantitative analysis of gene expression was done using the comparative CT (∆∆CT) method, normalizing expression of the gene of interest to Gapdh. The pre-validated Taqman gene expression assays for *Dll-1* (Mm01279269_m1), *Notch1* (Mm00435249_m1), *Notch2* (Mm00803077_m1), *Notch3* (Mm01345646_m1), *Notch4* (Mm00440525_m1), *Hes1* (Mm01342805_m1), *Jagged 1* (Mm00496902_m1), *Jagged 2* (Mm01325629_m1), *Dll-4* (Mm00444619_m1), *VEGF* (Mm01281449_m1) and control *Gapdh* (4352339E) (Applied Biosystems, Foster City, CA) were used. Real-time PCR was performed using the universal PCR master mix reagent (Applied Biosystems).

### Protein extraction

For protein extraction cells were sonicated in ice-cold 1X RIPA buffer (Santa Cruz Biotechnology) containing protease and phosphatase inhibitor (Roche Applied science, Indianapolis, IN). Lysates were incubated on ice for 30 min and centrifuged at 10,000 × g for 10 min at 4 °C. Supernatant fluid was collected and used as a total cell lysate for protein assays. Protein concentration was measured by BCA protein assay. Equal amounts of protein (50 μg) were used for ELISA.

### Immunofluorescence

The placenta and uterine horns were collected from control and treated groups. Tissues were fixed in 10% neutral-buffered formalin and processed as described previously^24^. 5-μm sections from frozen tissues were mounted onto silane-coated glass slides (Dako, Carpinteria, CA) and stored at −80 °C until used. The sections were submerged in sodium citrate buffer (pH = 6) and heated for 10 min in a microwave oven for antigen retrieval. For immunofluorescence sections were incubated with rat anti-F4/80 antibody, goat anti-DLL-1, rabbit anti-Notch1, rabbit anti-Hes1, rabbit anti-Jagged 1, rabbit anti-VEGF-receptor (Abcam, Cambridge, MA) or isotype-matched controls, overnight at 4 °C. Incubation with primary antibody was followed by secondary antibody: donkey anti-goat AF594, goat anti-rabbit FITC or rabbit anti-rat Dylight488 (Abcam). Cells were fixed in Prolong gold antifade reagent with DAPI (Invitrogen) to visualize the nuclei. Antigen distribution was examined using a Nikon Eclipse TE2000-S florescence microscope (Nikon Instrument INC, Melville, NY).

For semi-quantitative analysis of immunofluorescence levels, five squares with a fixed area (250X250 μm^2^) covering different regions of decidua or placenta were positioned. The mean integrated density value within a defined threshold was measured using image analysis software (Image J; NIH Image, National Institutes of Health, Bethesda, MD). Immunofluorescent measurements were obtained from a minimum of six decidual or placental sections and averaged. The average mean integrated density values (IDV) were plotted^53^.

### Cytokine/chemokine assay

A panel of mouse pro-inflammatory cytokines (TNF, IL-6, IFN-γ, IL-15 and IL-1α), chemokines (MIP-1β), anti-inflammatory cytokines (IL-10 and IL-13) and VEGF was analyzed either in medium from decidual macrophages or in total cell lysates from placental or decidual cells by Milliplex map kit (Millipore, St. Charles, MO) and assayed on a MAGPIX instrument (Millipore) as per the instructions provided by the manufacturer. Equal volume of medium or equal amounts of protein (50 μg) was used for the assay. The assay was repeated three times with duplicates.

### Statistical analysis

Continuous variables were assessed with two-tailed Student’s t-test or ANOVA. When data were not normally distributed, two groups were compared with Mann-Whitney U test. Categorical variables (e.g. preterm delivery) were analyzed using Fisher’s exact test or contingency tables. P < 0.05 was considered a statistically significant difference.

## Additional Information

**How to cite this article**: Jaiswal, M. K. *et al.* Notch Signaling in Inflammation-Induced Preterm Labor. *Sci. Rep.* **5**, 15221; doi: 10.1038/srep15221 (2015).

## Supplementary Material

Supplementary Information
