# Supplementary material for: Notch Signaling in Inflammation-Induced Preterm Labor
Source: Sci Rep. 2015 Oct 16;5:15221. doi: 10.1038/srep15221 (PMC4607997; doi:10.1038/srep15221)
Supplement: Supplementary Information [file srep15221-s1.doc]

**Supplementary information for the manuscript titled**

**Notch Signaling in Inflammation-Induced Preterm Labor**

**Mukesh K. Jaiswal^*,§,δ^, Varkha Agrawal^#,§^, Sahithi Pamarthy^*^,** **Gajendra K. Katara^*^, Arpita Kulshrestha^*^, Alice Gilman-Sachs^*^, Kenneth D. Beaman^*^ and Emmet Hirsch^#¶^,**

**Supplemental Figure 1:** (A) Representative image from uteroplacental unit from day 14.5 of pregnancy assessed by immunofluorescence. Original magnification: 25X. The demarcation of different zones is shown. M=myometrium, U=Uterine decidua and P=placenta. (B) Positive control: Flow cytometry analysis of RAW 264.7 macrophage cell line used as a positive control for the extraction of decidual macrophages from mouse decidua on day 14.5 of pregnancy. Red line=Isotype control; blue line=F4/80-APC antibody.


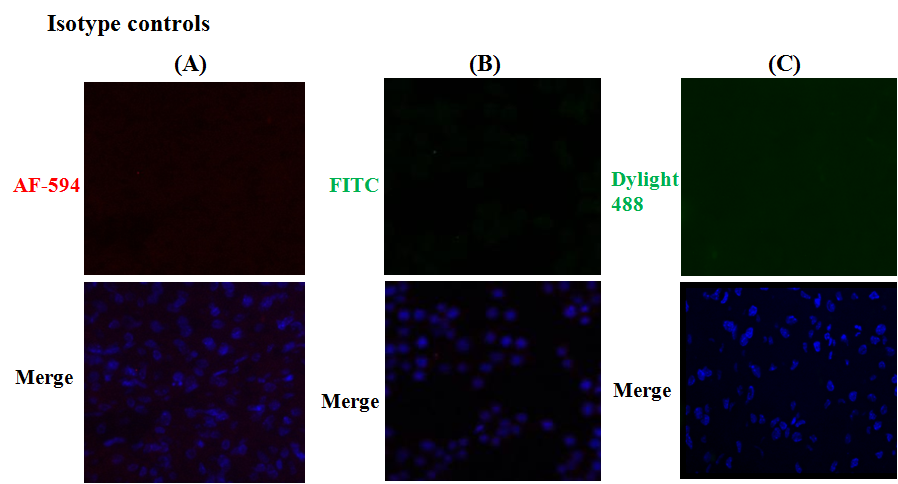
**Supplemental Figure 2:** Isotype controls for antibodies used in immunofluorescence studies and presented in the main paper figures:- (A) Goat IgG for DLL-1 (B) rabbit IgG for Notch1, Hes1 and Jagged 1 and (C) rat IgG for F4/80 antibody. Merged images with DAPI also shown. Original magnification: 200X.


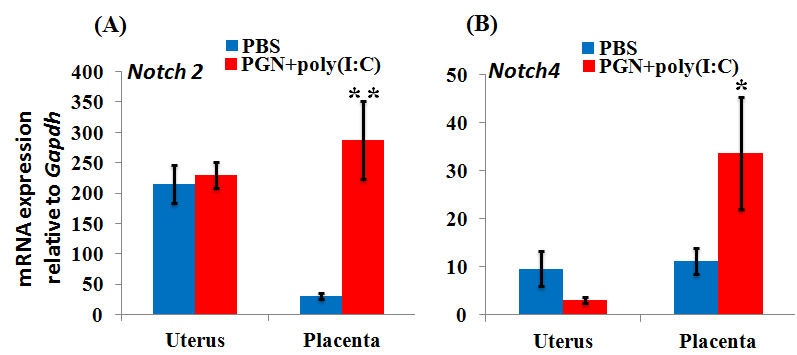


**Supplemental Figure 3: Notch receptor expression during PGN+poly(I:C)-induced preterm labor.** The mRNA expression of Notch2 (A) and Notch4 (B) in uterus and placenta recovered from PBS and PGN+poly(I:C) treated groups. N=6-11 each group. PBS and PGN+poly(I:C): intrauterine injections on day 14.5. Error bars=±SEM. *P≤0.05 **P≤0.01 Significant difference vs. PBS.


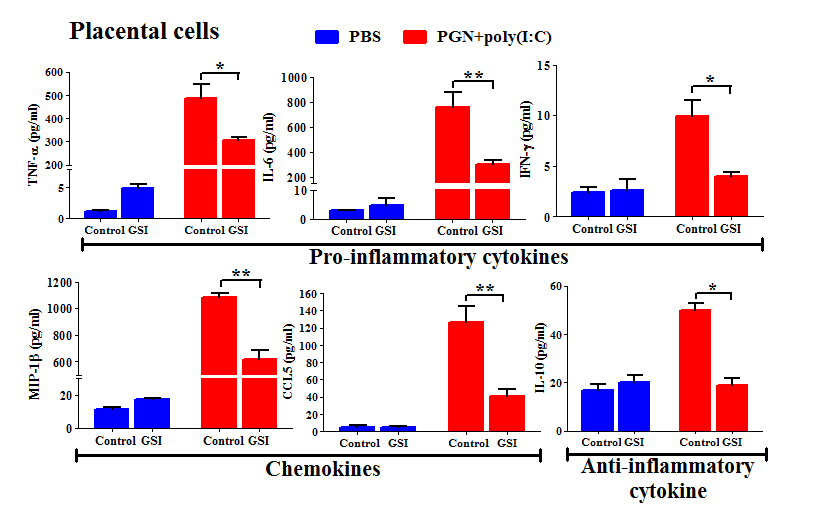


**Supplemental Figure 4: Inhibition of Notch signaling suppresses inflammatory responses in placental cells.** Pro-inflammatory and anti-inflammatory cytokines and chemokines were measured by Luminex assay in protein extracted from placental cells recovered from mouse on day 14.5 of pregnancy, cultured *ex vivo* and treated with PBS and PGN+poly(I:C) for 2h, followed by treatment with either control or GSI for 10h. N=3 each group. Error bars=±SEM. *P≤0.05, **P≤0.01 Significant difference between PGN+poly(I:C) treated with control/GSI.

**Supplemental Table 1: Effect of GSI on PGN+poly(I:C)**-**induced preterm delivery**

| **Treatment groups**  **Dose/mouse** | **Preterm delivery (%)** | **No. of pups alive *in-utero* after 48hrs** |
| --- | --- | --- |
| **PBS IU + Vehicle IU** | **0/3 (0)** | **12.00±0.57** |
| **PBS IU + GSI IU (300µg)** | **0/3 (0)** | **11.33±0.67** |
| **PGN+poly(I:C) IU + PBS IU (100µl)** | **3/3 (100)** | **0.33±0.33** |
| **PGN+poly(I:C) IU + Vehicle IU** | **7/7 (100)** | **0.9±0.62** |
| **PGN+poly(I:C) IU + GSI IU (300µg)** | **4/9 (44.5%)*** | **7.9±1.23**** |

*P≤0.05, **P≤0.01 Significant difference vs. PGN+poly(I:C) IU + Vehicle IU
